# COVID-19 Infection in Vaccinated Healthcare Professionals

**DOI:** 10.7759/cureus.23386

**Published:** 2022-03-22

**Authors:** Muneeb Ullah, Muaz Mubashir, Hassan Atique, Farhan Aslam, Musfirah Tahir, Mehdi Naqvi

**Affiliations:** 1 General Surgery, Maroof International Hospital, Islamabad, PAK; 2 Internal Medicine, Federal Government Polyclinic Hospital, Islamabad, PAK; 3 Paediatrics, Maroof International Hospital, Islamabad, PAK

**Keywords:** healthcare workers, breakthrough infections, healthcare professionals, covid-19 vaccination, covid 19 infection

## Abstract

Background: There are different types of Coronavirus disease (COVID-19) vaccines available presently, and their emergency use has been approved by WHO worldwide on a mass scale. COVID-19 vaccine breakthrough infections have been reported worldwide. In Pakistan, there are limited data on COVID-19 vaccine breakthrough infections and their clinical course, especially in healthcare professionals (HCPs). Our study aims to investigate COVID-19 infections among vaccinated HCPs.

Methods: A prospective study was conducted on 425 healthcare professionals. Data collected from healthcare professionals included names, age, gender, number of vaccination doses, COVID-19 infection pre and post-vaccination, the severity of COVID-19 infection (if positive), and co-morbid conditions. Ethical board approval was taken. Statistical Package for Social Sciences (SPSS) version 23 (IBM Corp., Armonk, NY) was used to analyze the data.

Results: After complete vaccination, 17.27% acquired COVID-19 infection; 2.47% had COVID-19 infection both pre and post-vaccination. The mean age was 32.46 years (n=71) with a standard deviation of ±9.376. The male to female ratio was 1.53. COVID-19 PCR was positive in 95.77%. During the course of the disease, 4.2% were asymptomatic, 92.95% had mild symptoms, 1.4% were hospitalized, and 1.4% had to be managed in the intensive care unit. None of the HCPs who had received booster doses acquired a COVID-19 infection.

Conclusion: It was found that prior COVID-19 infection and vaccination do not confer immunity from infection. However, proper vaccination limits the severity, morbidity, and mortality of COVID-19 infection.

## Introduction

There are different Coronavirus disease 19 (COVID-19) vaccines available presently. Their emergency use is approved by the World Health Organization (WHO) worldwide on a mass scale [1]. Clinical trials on licensed vaccines showed efficacy ranging from 70% to 95% against COVID-19 infection [2-5]. However, COVID-19 breakthrough infections have been reported among vaccinated individuals [6-8]. Healthcare professionals (HCPs) belong to a high-risk group of individuals due to their continuous or recurrent exposure to COVID-19 infected individuals and the environment. COVID-19 vaccine breakthrough infections range from 3% to 17% among HCPs [9]. In Pakistan, there are limited data on COVID-19 infections after vaccination and their clinical course. The aim of this study is to investigate the presence of COVID-19 infections among vaccinated HCPs and their severity.

## Materials and methods

A prospective study was performed from November 2021 to January 2022 with a total of 425 HCPs. HCPs included doctors, nursing staff, hospital administrators, ward boys, sanitary workers, etc. A proforma was designed to collect data from individuals face-to-face, and the same proforma was used to collect data via an online survey. Collected data included name, age, gender, number of vaccination doses, COVID-19 infection that occurred pre and post-vaccination, the severity of COVID-19 infection (if positive), and co-morbid conditions. The presence of COVID-19 infection was confirmed through PCR testing, rapid antigen testing, and high resolution computed tomography (HRCT) of the chest. The severity of the disease was ascertained through a combination of oxygen requirement, clinical symptoms, HRCT findings, and laboratory parameters. All the HCPs who were tested either had a recent exposure (within a week) to a COVID-19 infected individual or had symptoms. Those who were infected within three weeks after the second dose of vaccination were excluded from the study and were not considered part of the breakthrough infections after vaccination. Patient confidentiality was maintained. Ethical board approval was obtained from the Federal Government Polyclinic Postgraduate Medical Institute, Islamabad. The Statistical Package for Social Sciences (SPSS) version 23 (IBM Corp., Armonk, NY) was used to analyze the data.

## Results

**Figure 1:**
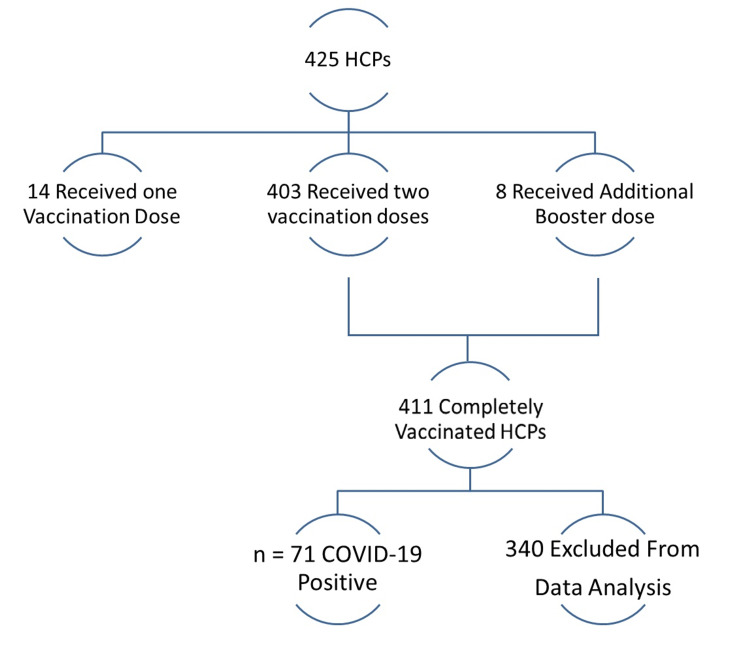
Flowchart regarding patient selection

A total of 425 HCPs were included in the study. Fourteen (3.3%) had received only a single dose of vaccination and were excluded from further analysis; 403 (94.8%) had received a minimum of two doses of vaccination, while eight (1.9%) HCPs had received an additional booster dose. Out of these fully vaccinated 411 HCPs, 71 (17.27%) acquired COVID-19 infection after complete vaccination. Data analysis was performed on these 71 (n) HCPs as shown in Figure 1.

The mean age was 32.46 years, with a standard deviation of ±9.376. The minimum age was 20 years and the maximum was 61 years. Out of these, 43 (60.6%) were males and 28 (39.4%) were females. The duration after vaccination when the COVID-19 infections occurred in HCPs is depicted in Table 1.

**Table 1:** Duration after vaccination when COVID-19 infection occurred

Number (n = 71)	Frequency %	Duration after vaccination
17	23.9	3 weeks to 2 months
17	23.9	2 months to 3 months
8	11.3	3 months to 4 months
15	21.1	4 months to 5 months
6	8.5	5 months to 6 months
6	8.5	6 months to 7 months
2	2.8	7 months and onwards

Eleven (2.47%) of the total (n=71) had COVID-19 infection both pre and post-vaccination. COVID-19 PCR was positive in 68 (95.77%) HCPs and was the most common test. COVID-19 antigen was positive in one (1.4%) HCP, while HRCT was used to diagnose positivity in four HCPs (5.6%). During the course of the disease, 66 (92.95%) had mild symptoms while 3 (4.2%) were asymptomatic, as shown in Table 2.

**Table 2:** Severity of COVID-19 infection after vaccination

Disease severity	Number (n = 71)	Frequency %	Vaccination doses
Asymptomatic	3	4.2	2
Mild symptomatic	66	92.95	2
Hospitalized	1	1.4	2
Intensive care unity stay	1	1.4	2
Ventilator support	None	None	2
Mortality	None	None	2

None of the HCPs who had received booster doses acquired COVID-19 infection. Two had diabetes and hypertension while only one had each asthma and hyperthyroidism.

## Discussion

The available vaccines are very important in the fight against COVID-19 infection, and they work on the basis of generating an immune response that creates antibodies and cells that result in protecting against COVID-19 infection [10]. Despite complete vaccination, a small proportion of individuals will contract COVID-19 infection since no vaccine accords 100% protection against the disease [11]. In our study, 17.27% of HCPs had COVID-19 infection after vaccination. This shows that a significant proportion of the population is still at risk of infection after vaccination. This percentage is higher as compared to another local study that showed 0.31% of HCPs were infected with COVID-19 after vaccination [1]. This, however, correlates well with a study (14.28%) conducted in India [12]. This can be a consequence of breach in precautionary measures post vaccination, associated with the recent surge secondary to newer variants or more prolonged follow-up of patients after vaccination. Vaccines that were produced for the initial strain of SARs-CoV-2 had less efficacy against the newer variants, which may also be a contributing factor [13]. Male dominance of 60.6% was seen in our study while the mean age was 32.46 ± 9.376 years. These percentages were close to those of a study conducted in India [14]. Our results show that 97.18% of HCPs suffered from asymptomatic to mild infections, which is also seen in other studies [1,15]. One HCP was admitted to the intensive care unit but did not require ventilator support. Our study reports no mortality, as was seen in a local study [1]. This was also comparable to regional studies in India [13,16]. This shows that vaccination has overall decreased the severity of the disease. Only 1.9% of the vaccinated HCPs had received additional booster doses while our study was being performed. None of the HCPs who had received booster doses developed an infection. This needs further data to correlate the outcome after booster doses. 2.47% of those who had infections after vaccination were also infected with COVID-19 infection prior to vaccination [17]. Thus, prior COVID-19 infection and vaccination do not completely confer immunity against the virus. After the second dose of vaccination, 23.9% were infected within two months, another 23.9% after two months, 11.3% after three months, 21.1% after four months, 8.5% after five months of vaccination, and 2.8% after seven months. The study duration was short, which could have been affected by climatic changes, newer variants, types of vaccines available, etc. The data on COVID-19 infection after vaccination duration are limited and need to be evaluated further as our study showed low frequencies as the duration increased, especially after five months. The changing trends of COVID-19 infections, the study population, the efficacy of different vaccines, their cold chain maintenance, booster doses, the emergence of variants, different waves, demographics, and regional differences warrant further studies [18].

## Conclusions

It was seen that previous COVID-19 infection does not confer immunity from infection, which was confirmed through PCR tests, rapid antigen tests, and HRCT. Vaccines cannot give full immunity against COVID-19 infection as yet, but they limit the severity, morbidity, and mortality of COVID-19 infection. Booster doses of the vaccine show promising data and should be considered as important as initial vaccination itself.
